# Comparison of Interlaminar and Transforaminal Approaches for Treatment of L_5_/S_1_ Disc Herniation by Percutaneous Endoscopic Discectomy

**DOI:** 10.1111/os.12831

**Published:** 2020-12-03

**Authors:** Aiguo Gao, Huilin Yang, Liyan Zhu, Zhangjie Hu, Binbin Lu, Qi Jin, Ye Wang, Xiaofeng Gu

**Affiliations:** ^1^ Department of Orthopaedics Wuxi People's Hospital Affiliated to Nanjing Medical University Wuxi City China; ^2^ Department of Orthopaedics The First People's Hospital Affiliated to Suzhou University Suzhou City China

**Keywords:** Clinical efficacy, Lumbar disc herniation, Minimally invasive spine surgery, PEID, PETD

## Abstract

**Objective:**

The aim of this study was to compare the clinical efficacy of percutaneous endoscopic interlaminar discectomy (PEID) and percutaneous endoscopic transforaminal discectomy (PETD) in treating L_5_/S_1_ disc herniation.

**Methods:**

A retrospective analysis of 76 patients with L_5_/S_1_ intervertebral disc herniation was performed. There were two surgical treatment groups: one with patients receiving PEID and the other with patients receiving PETD. The two groups were compared by length of surgery, times of intraoperative X‐ray exposure, postoperative time in bed, length of hospital stay, operative complications, patient's assessment of pain using a visual analogue scale (VAS), and disability using the Oswestry disability index (ODI) before and after surgery.

**Results:**

Subjects in the PEID group were in surgery for 60.90 ± 13.11 min and needed intraoperative X‐ray exposure 4.10 ± 1.09 times. Patients in this group were ambulatory by 7.52 ± 1.08 h after surgery and were hospitalized for 5.05 ± 0.92 days. In contrast, patients in the PETD group were in surgery for 84.06 ± 15.58 min and needed intraoperative X ray exposure 12.81 ± 8.46 times. These patients were ambulatory by 7.06 ± 0.91 h after surgery and remained in the hospital for 4.94 ± 0.80 days. Based on these data, operation time and fluoroscopy time were significantly less (*P* < 0.002 and *P* < 0.001, respectively) for subjects in the PEID group. However, ambulatory time and hospitalization were similar for both in terms of pain relief and decreased disability, and subjects in both groups responded well to the surgery and showed a significant decrease in both VAS and ODI scores at their 1‐year follow‐up (*P* < 0.01). Furthermore, there were no statistically significant differences between the two surgeries in terms of pain relief and decrease in disability.

**Conclusion:**

For L_5_/S_1_ disc herniation, PEID and PETD provide similar results for patients. However, PEID has the advantage over PETD in that it is a shorter procedure and exposes the patient to less radiation.

Keywords

## Graphical abstract

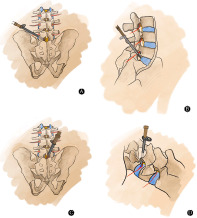

## Introduction

Lumbar disc herniation (LDH) is a common disease in spine surgery. It has the characteristics of obvious hyperplasia of local tissue, heavy degeneration of intervertebral discs, and long course of disease. The cause of LDH is related to the degenerative changes of different degrees caused by external forces on various parts of the lumbar disc, finally pressing or stimulating the adjacent spinal nerve roots^1^, ^2^. Lumbocrural pain is a common clinical symptom of this disease. Early application of traditional open surgery for LDH has a good curative effect, but the surgical trauma is considerable, postoperative complications are significant, and the postoperative recovery time is long^3^, ^4^. With the continuous improvements in surgical technology, percutaneous intervertebral foramina technology has become an effective scheme in the field of minimally invasive spinal surgery for LDH. Many studies have shown that this technique has the same curative effect as traditional open surgery. It has the advantages of small incision, small soft tissue injury, less bleeding during the operation, quick recovery after the operation, early activity, relatively low hospitalization cost, and a low wound infection rate. There are two main types of percutaneous discectomy, percutaneous interlaminar endoscopic discectomy (PEID) and percutaneous transforaminal endoscopic discectomy (PETD), each of which has a comparative advantage in the treatment of disc herniation.

The lumbar spine at the level of L_5_/S_1_ has several anatomic characteristics, including high iliac crest blockage, relatively narrow foramen, and wide interlaminar distance, which bring technical challenges to PETD performance at the L_5_/S_1_ level. Therefore, PEID is opted for the treatment of L_5_/S_1_ disc herniation. However, Yeung *et al*.^5^ demonstrated that PETD can be successfully used for treatment of all lumbar levels, including L_5_/S_1_. The relative advantages of PEID over PETD or vice versa remain controversial in the treatment of L_5_/S_1_ disc herniation.

As far as we know, there are few studies comparing the clinical efficacy and safety of PEID and PETD. The present paper reports the clinical results of a retrospective comparative study of percutaneous endoscopic treatment of lumbar disc herniation in our department from January 2016 to December 2018. By comparing the therapeutic effects of PEID and PETD in the treatment of L_5_/S_1_ disc herniation, we hope to achieve the following: (i) to investigate the clinical efficacy of two surgical approaches; (ii) to make clear the advantages of the two surgical approaches; and (iii) to provide some basis for clinical selection of surgical approaches.

## Methods

### *Inclusion and Exclusion Criteria for the Study*

This study was approved by the ethics review committee of Wuxi People's Hospital Affiliated to Nanjing Medical University. The inclusion criteria were: central, paracentral, or prolapsed L_5_/S_1_ disc herniation and failure of formal conservative treatment. The exclusion criteria were: intervertebral disc inflammation or tuberculosis, recurrent disc herniation, multiple segments of disc herniation, lumbar instability, such as lumbar spondylolisthesis, widely lumbar stenosis, and far lateral lumbar disc herniation.

### *Subjects*

A retrospective analysis was performed on surgical patients seen in the Wuxi People's Hospital from January 2016 to December 2018 for L_5_/S_1_ single segment lumbar disc hernias. A total of 76 eligible patients were enrolled in the study. Among these, 38 were treated with PEID; in this cohort, there were 18 men and 20 women, aged 24 to 72 years (average, 39.53 ± 9.91 years). In the second group, there were 38 subjects treated with PETD; in this cohort, there were 21 men and 17 women, aged 19 to 88 years (average, 41.12 ± 10.12 years). Before surgery all patients provided consent to participate in the study and received preoperative lumbar spine X‐rays, dynamic position X‐rays, and CT or MRI examinations to definitively confirm their diagnosis and exclude the presence of other spinal diseases. The baseline data of the two groups are shown in Table 1. There are no significant differences between the two groups. All operations were performed by the same surgeon and all surgical instruments were obtained from Joimax GmbH (Karlsruhe, Germany). The surgical instruments obtained from Joimax GmbH include intervertebral foramen and corresponding spinal minimally invasive surgical instruments, imaging and image processing systems, and an Ellman dual frequency radio frequency machine.

**TABLE 1:** Comparison of radix data between the two different approaches

Group	Number of cases	Gender		Age (year)	BMI
		Male	Female		
PEID	38	18	20	39.53 ± 9.91	23.19 ± 4.47
PETD	38	21	17	41.12 ± 10.12	24.80 ± 4.38
*t*/*x* ^2^		0.523		0.473	0.259
*P*‐value		0.471		0.596	0.451

Values are presented as the mean ± SD unless otherwise indicated.

BMI, body mass index; PEID, percutaneous endoscopic interlaminar discectory; PETD, percutaneous endoscopic transforaminal discectomy.

### *Surgical Methods*

#### *Percutaneous Endoscopic Interlaminar Discectomy Group*

##### Posture and Positioning

Patients were placed in the prone position on a bow‐type frame with the operating bed positioned so that the bow in the patient's back was minimized to expand and open the interlaminar window. The side view of the C‐arm was used to determine the operative segment. After injection of local anesthetic, the positioning needle was inserted around 2.0 cm from the median line after the corresponding segment.

##### Establishment of Working Channel

**Fig 1:**
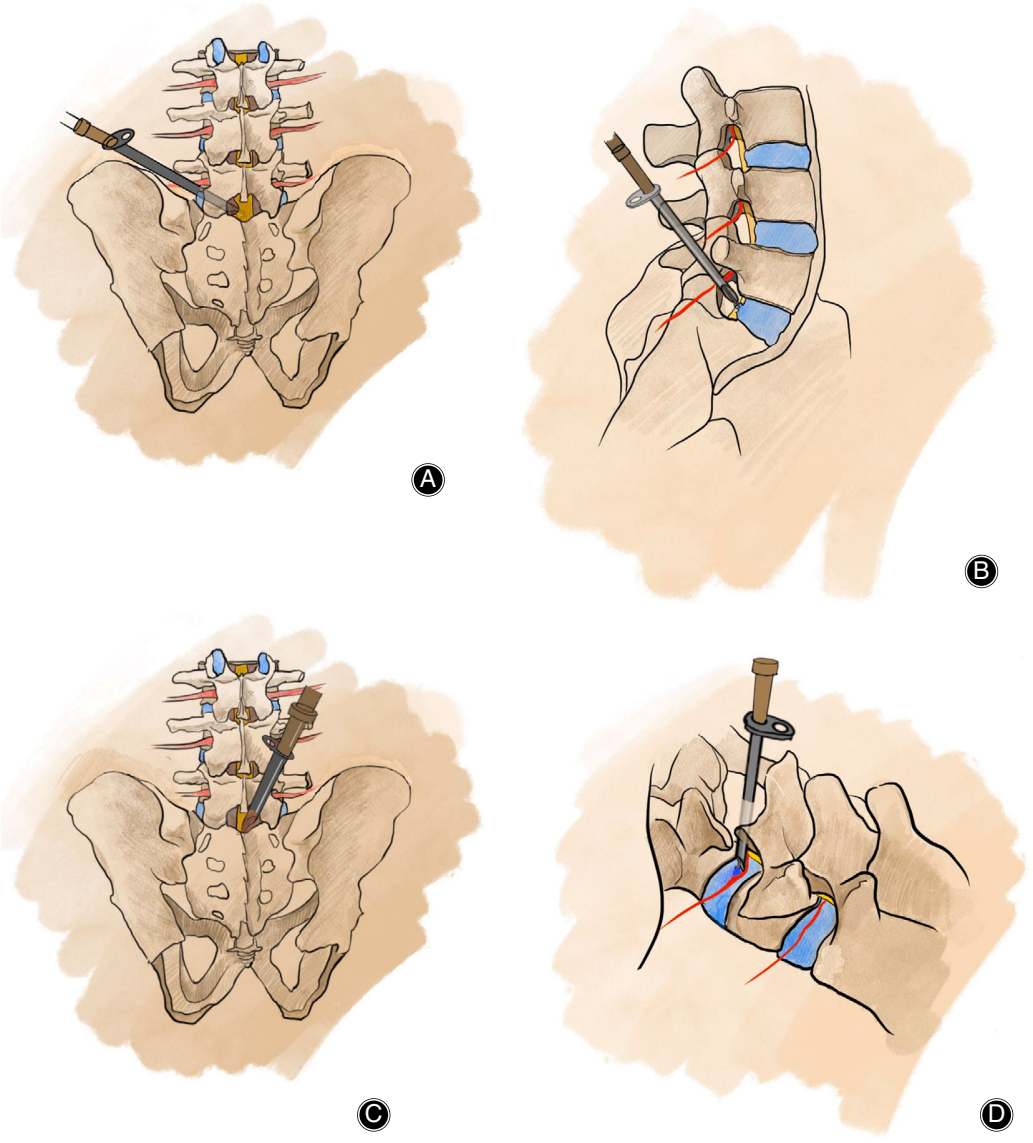
(A, B) Schematic diagram of percutaneous endoscopic transforaminal discectomy. (C, D) Schematic diagram of percutaneous endoscopic interlaminar discectomy.

A 5‐mm longitudinal incision was made along the side of the spine adjacent to the operative segmental and the deep fascia was incised. The extension tube of the pencil head was placed into the superficial yellow ligament, close to the root of the spine. Next, the expansion tube was placed into the work pipeline and the C‐arm was repositioned to determine its position. The expansion tube was then retracted into the endoscope (Fig. 1A).

##### Endoscopic Operation

All fibrous adipose tissue on the surface of the yellow ligament was cleaned with medullary forceps. The work pipeline was then parallel to the yellow ligament and the medial border of the joint was inserted into the spinal canal. From this position, a prominent collapsed nucleus pulposus could be seen, along with nerve root compression, exposed epidural fat, the nerve root, and the epidural sac. Then the work pipeline was adjusted so that the nerve root could be probed. The nerve root was exposed to the root and then relieved by the nerve strip. Next the disc was exposed and the nucleus pulposus removed by scraping laterally back and forth and removing the adjacent degenerated medullary nucleus. The endoscope was then adjusted to observe the S_1_ region of the nerve root to ensure that degenerated tissue was completely removed and that S_1_ nerve root activity was intact and decompression complete. When the procedure was complete, the Joimax radio frequency electrode line fiber ring configuration was removed and unplugged, and the surgical site was covered with sterile dressing.

#### *Percutaneous Endoscopic Transforaminal Discectomy Group*

##### Posture and Positioning

Patients were placed in the prone position on a bow‐type frame that was solidly attached to the operating table to widen the interlaminar window. The median line of the lateral iliac spine was located along the median line of the spine. The horizontal line in the middle of the intervertebral space was demarcated by the X‐ray fluoroscopy of the c‐type arm. The intersection of this line and the posterior midline was at the center of the intervertebral disc. Along the side of the posterior median line, 12–14 cm was used as a parallel line, and then the lateral position line of the center of the intervertebral space was demarcated in the oblique direction of the lateral position X‐ray. The intersection point of the two lines was the insertion point.

##### Establishment of Working Channel

One percent lidocaine was used for the puncture site. According to the calibration and the waist is 25° to 30° skin surface puncture, C‐arm fluoroscopy guided under the needle puncture direction and point of view, the whole infiltration anesthesia, when the puncture needle point on the X line is a perspective on the pedicle midpoint wired up and down, side perspective on the attachment is located in the upper and lower vertebral rear, puncture needle which reach Kambin security triangle intervertebral disc trailing edge. The needle was used to puncture the central disc. A mixture of alcohol, methylene blue, and iodine from sea salt (volume ratio 1:1) was injected. Using disk imaging, the needle was removed after being threaded again. After local anesthesia, a 5 mm long longitudinal incision was cut on the skin, and the guide rod was inserted into the posterior edge of the intervertebral disc under fluoroscopy guidance. At this point, the guide bar was located on the line of the spinous process under the positive perspective, and the lateral perspective was located on the posterior 1/3 of the intervertebral disc. The guide wire was taken out and rotated through the guide bar, step by step. The work sleeve was then inserted into the intervertebral disc. In the process, if the joint is blocked, a TESSYS special ring saw can be used to remove the hyperplastic bone and part of the articular process, so that the intervertebral opening is enlarged (Fig. 1B).

##### Endoscopic Operation

The pencil‐head expansion tube was taken out and the endoscope was placed in the work sleeve. Microscopically, the degenerative nucleus pulposus tissue could be seen under microscope. The nucleus pulposus was removed with different types of nucleus pulposus forceps. By rotating the working sleeve and rotating backward, the nucleus pulposus tissue in the spinal canal was removed, and the adhesion of scar tissue around the nerve root was properly removed to ensure adequate decompression. The Joimax dual‐frequency radiofrequency electrode ablated the flocculated nucleus, cauterized the fibrous ring, and controlled the hemorrhage around the spinal canal and the nerve roots with the radiofrequency electrocoagulation. The field was rerinsed with water, the work sleeve was pulled out, and then the site was covered with sterile dressing.

### *Assessment of Clinical Efficacy*

For the two surgical procedures, the duration of surgery, times of intraoperative X‐ray exposure, time to first post‐surgical ambulation (after recovery from anesthesia), and incidence of post‐surgical complications were assessed. Subjective assessment of pain, before and after surgery and at follow‐up, was evaluated using the visual analogue scale (VAS), while disability (at the same visit) was assessed using the Oswestry disability index (ODI). Anteroposterior and lateral lumbar X‐rays were obtained on the day of surgery. At 1, 3, 6, and 12 months after surgery, CT examination of lumbar intervertebral discs was performed.

### *Statistical Analysis*

Statistical analysis was performed using statistical software SPSS version 19.0 (SPSS, Chicago, IL, USA). All data except for gender were expressed as the mean ± standard deviation (SD). Gender proportion was compared between PEID and PETD using χ^2^ analysis and independent *t*‐tests for categorical and continuous variables, respectively. Statistical significance was set at *P* < 0.05.

## Results

A retrospective analysis was performed on 76 patients with L_5_/S_1_ intervertebral disc herniation who were admitted to Wuxi People's Hospital from January 2016 to December 2018. There were two surgical treatment groups: patients receiving PEID and those receiving PETD. Both groups of patients successfully completed the surgeries and there were no serious complications (e.g. positioning errors, catheter tears, or nerve root injury). Furthermore, no postoperative infections or cases of poor wound healing were observed.

### *Statistical Results*

Subjects in the PEID group were in surgery for 60.90 ± 13.11 min and needed intraoperative X‐ray exposure 4.10 ± 1.09 times (Table 2). Patients in this group were ambulatory by 7.52 ± 1.08 h after recovery from anesthesia and were hospitalized as inpatients for 5.05 ± 0.92 days. In contrast, patients in the PETD group were in surgery for 84.06 ± 15.58 min and needed intraoperative X‐ray exposure 12.81 ± 8.46 times. These patients were ambulatory by 7.06 ± 0.91 h after recovery and remained in the hospital for 4.94 ± 0.80 days. Based on these data, operation time and fluoroscopy time were significantly less (*P* < 0.002 and *P* < 0.01, respectively) for subjects in the PEID group compared to the PETD group. However, there were no statistically significant differences between the two groups for ambulatory time and time in the hospital.

**TABLE 2:** Clinical assessment of subjects, before and after surgery, in the two treatment groups

Group	Number of cases	Duration of surgery (min)	Times of intraoperative X‐ray exposure	VAS score		Time to first post‐surgical ambulation (h)	Hospital stay (days)
				Preoperative	Last follow‐up		
PEID	38	60.90 ± 13.11	4.10 ± 1.09	7.19 ± 0.98	1.33 ± 0.73	7.52 ± 1.08	5.05 ± 0.92
PETD	38	84.06 ± 15.58	12.81 ± 8.46	7.22 ± 0.96	1.25 ± 0.80	7.06 ± 0.91	4.94 ± 0.80
*t‐*value		5.624	12.989	0.103	0.383	1.674	0.461
*P*‐value		<0.001	<0.001	0.918	0.083	0.100	0.646

Values are presented as the mean ± SD unless otherwise indicated.

PEID, percutaneous endoscopic interlaminar discectory; PETD, percutaneous endoscopic transforaminal discectomy; VAS, visual analogy score.

### *Treatment Effect*

**Fig 2:**
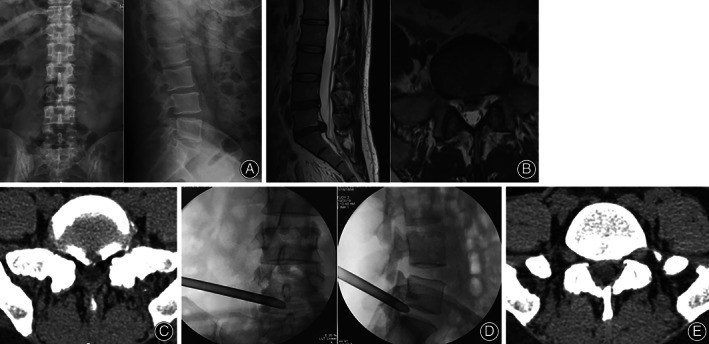
Woman, 45 years old. Main complaint: lateral pain of right leg with numbness for half a year. (A) Preoperative X‐ray films. (B) Preoperative MRI shows that L_5_S_1_ disc herniation and the protruding nucleus pulposus compresses the dural sac. (C) Preoperative CT image shows L_5_S_1_ disc herniation. (D) The position of the working cannula using the intervertebral foramen approach. (E) CT image shows that the nucleus pulposus was completely removed.

**Fig 3:**
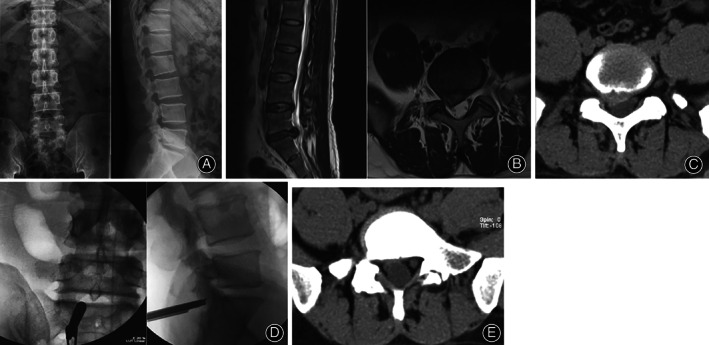
Male, 42 years old. Main complaint: lumbago for 3 years, with pain and numbness of right lower extremity for 1 month. (A) Preoperative X‐ray films. (B) Preoperative MRI showed L_5_S_1_ disc herniation. (C) Preoperative CT showed L_5_S_1_ disc herniation. (D) The position of the working cannula through the inter lamina approach during the operation. (E) CT image after operation shows that the herniated intervertebral disc has been taken out.

In terms of pain and disability, subjects in the PEID group had average VAS scores of 7.19 ± 0.98 before surgery. The scores dropped to 1.33 ± 0.73, respectively, at 1‐year follow‐up (Table 2). Similarly, patients in the PETD group had average VAS scores of 7.22 ± 0.96, falling to 1.25 ± 0.80, respectively, after 1 year. Irrespective of the treatment group, subjects showed a significant improvement in both assessments at final follow‐up (*P* < 0.01). Furthermore, there was no statistically significant difference between the two surgeries in terms of pain relief and decrease in disability (*P* > 0.05). Postoperative ODI indicated that there was significant improvement in leg and waist pain in both groups when compared with preoperative scores (*P* < 0.01) (see Figs 2 and 3). There was no significant difference in ODI scoring between the two groups either preoperatively or postoperatively (*P* > 0.05) (Table 3).

**TABLE 3:** Comparison of preoperative and postoperative ODI in the two treatment groups

Group	Number of cases	ODI score (%)				
		Preoperative	1 month after operation	3 months after operation	6 months after operation	12 months after operation
PEID	38	68.02 ± 15.77	47.48 ± 15. 66	31.72 ± 7.55	19.88 ± 7.68	14.55 ± 7.34
PETD	38	67.85 ± 16.57	49.14 ± 14. 85	30.59 ± 9.64	19.17 ± 8.26	15.81 ± 6.28
*t‐*value		0.725	0.509	0.671	0.801	0.691
*P*‐value		0.681	0.241	0.714	0.199	0.593

Values are presented as the mean ± SD unless otherwise indicated.

ODI, Oswestry disability index; PEID, percutaneous endoscopic interlaminar discectory; PETD, percutaneous endoscopic transforaminal discectomy.

Although relatively rare, both treatment groups had two patients with postoperative nerve root pain and one patient in each with recurrence. In both cases, the patients responded well to standard management approaches.

## Discussion

### *Status of Research in the Field*

The lumbar spine is an important part of the human body and is actively engaged in many routine “daily activities.” Repeated exertion, weight‐bearing, and other activities can accelerate the degeneration of the lumbar spine and cause symptoms such as low back pain, radiating pain, numbness, and weakness in the lower extremities. More frequently, the incidence of LDH is greater than 90% in L_4/5_ and L_5_/S_1_ intervertebral discs at the junction of the spine and pelvis, because these joints are more prone to degeneration and damage than the other lumbar disks^1^, ^2^.

Treatment of lumbar disk degeneration is divided into two stages. The first stage is a conservative treatment and routinely includes anti‐inflammatory and analgesic medications, bed rest, and various physical measures (e.g. hot or cold compresses and physical therapy). When conservative treatment fails to bring relief, surgical treatment is then considered. Surgery is divided into open and minimally invasive procedures based on the size of the incision needed to access the area and the level of trauma present. Although the development of these technologies was exciting and revolutionary for the treatment of L_5_/S_1_ disk herniation, the steep learning curve necessary to master the approach through the intervertebral foramen slowed its implementation by surgeons. Difficulties related to the procedure, especially for those new to the technique, resulted in increased time in the operating room, increased exposure of the patient to X‐rays, and failure to gain access to (i.e. inability to puncture) the intervertebral disk due to obstruction of the iliac crest.

In 2008, Ruetten *et al*.^6^ were the first to describe the use of an endoscope for performing PEID. The approach was particularly well adapted for patients with a high iliac crest and L_5_ transverse process because the L_5_/S_1_ interlaminar space is wider and provides more favorable anatomical access to perform the discectomy^7^, ^8^.

### *Comparison of the Two Surgical Approaches*

The results of this study showed that there are no significant differences in the efficacy of the two surgical procedures as the patients had equivalent length of time in bed before becoming ambulatory, length of hospitalization, incidence of complications, recurrence rates, and improved postoperative VAS and ODI scores. Furthermore, the therapeutic effect of PEID for L_5_/S_1_ lumbar disc herniation was equivalent to that of PETD. Both PEID and PETD have been shown here and in other studies to be safe and effective minimally invasive treatments for L_5_/S_1_ lumbar disc herniation.

Although the efficacy of the two surgical techniques is similar, they have different characteristics while being performed. In this study, the fluoroscopy time for subjects in the PETD group was significantly longer than for those in the PEID group, indicating that puncture of the L_5_/S_1_ joint was more difficult. Often, this time was several times longer than for those in the PEID group. In the PETD group, access to the L_5_/S_1_ joint was obstructed in some patients due to the presence of a high iliac crest and joint process accretion, resulting in the use of a steep angle^3^. In addition, puncture and catheterization of the joint were relatively difficult due to obesity and other factors that limited access. Some of the patients needed the L_5_/S_1_ joint expanded, due to stenosis of the intervertebral foramen, which greatly increased the operating time and radiation exposure. By comparison, the PEID approach typically only required two or three rounds of fluoroscopy.

### *Choice of the Two Surgical Approaches*

Percutaneous endoscopic transforaminal discectomy is the approach of the intervertebral foramen. The local anatomy of the intervertebral foramen has a certain influence on its puncture and catheterization. Its indications are: central type, paracentral type, extreme lateral type, and prolapse free type. The relative contraindications are high iliac crest block, transverse process variation and hypertrophy block^6^. The PEID approach is clear in tissue level, and the puncture needle can reach the ligamentum flavum after passing through the skin, the superficial fascia, and the vertical spinal muscle. Only the ligamentum flavum is covered at the entrance of the lamina space; there is no important nerve passing through, there is no transverse process and iliac crest blocking, the puncture requirements are greatly reduced, and there is no damage to the facet joint process or iatrogenic lumbar instability. The indications are: paracentral type and the height of the nucleus pulposus^7^. However, for patients with extreme lateral protrusion, it is extremely difficult to puncture, which is the relative contraindication of PEID. The choice of approach for prolapse patients depends on the degree of disc prolapse and the skill of the operator. Generally, for L_4/5_ and L_5_/S_1_ patients with slight prolapse, both approaches are available. Before the operation, the size of the intervertebral foramen was evaluated by lumbar digital radiography (DR). The intervertebral foramen was obviously narrow, which could not meet the needs of catheterization or puncture. The PEID was used for L_5_/S_1_ patients with a high iliac crest, transverse process hypertrophy, and other bone structures that were difficult to block the operation of PETD. The PEID was selected for L_5_/S_1_ patients with severe prolapse, L_3/4_ and high disc prolapse, and recurrent LDH. The advantage of PETD is that the previous scar tissue of PEID affects the operation under the microscope but PETD is not affected by this.

### *Surgical Experience*

Based on the results presented here, we believe that PEID has some significant advantages over PETD for treating L_5_/S_1_ lumbar disk herniation. PEID eliminates the need to gain access to the joint blocked by the iliac crest, provides rapid identification of a puncture site, has a short operation time, and requires less exposure to radiation during the operation. Indications for the use of PEID in treating L_5_/S_1_ lumbar disc herniation are: central type, and, in particular, paraspinal central type and free prolapse type^9^, ^10^. Although there are some difficulties in using PEID for the central type, access to the center of the spinal canal to resect the protruding nucleus pulposus can be gained near the axillary entry of the nerve root or by increasing the angle of inclination of the endoscopy tubes. In this case, during surgery, special attention must be paid to gently reducing the risk of tearing the dura mater spinalis.

One of the biggest problems faced by surgeons using PETD is the puncture and the difficulty in accomplishing this efficiently, which has a fairly steep learning curve^11^. All imaging data for the patient must be carefully evaluated before beginning the operation so that the correct needle point can be selected ahead of time. The intervertebral foramina must be expanded so that the working catheter can be placed when the articular facet is blocked during the puncture. This process requires the repeated use of fluoroscopy and increases the likelihood of more lengthy surgical time, increased radiation exposure, and postoperative lumbar instability^12^. Moreover, because of the smaller intervertebral foramina in L_5_/S_1_, the nucleus pulposus collapses more when it becomes degraded and loses water, resulting in the narrowing of the intervertebral space. Hypertrophy of the articular facet joint in some patients makes the operating window of the intervertebral foramen more narrow^13^. Because of this, patients undergoing PETD have a more prolapsed nucleus pulposus, which may not be completely removed; this results in tissue being left after the operation, which compromises the effectiveness of the operation^14^. However, with PEID, access is gained to the canalis spinalis through the interlaminar space. During this procedure, the L_5_/S_1_ joint space is relatively wide and the working conduit is more freely mobile, which provides increased access to the nucleus pulposus tissue protruding outside the plane of the disc^15^.

Although PETD is more difficult than PEID in surgical operations and has higher requirements for operators, PETD has some unique advantages: (i) the original structures such as the ligamentum flavum in the lumbar posterior column are retained, with less trauma, through the intervertebral foramina approach^16^; (ii) for patients with recurrent LDH, PETD is the best choice, because PEID is more likely to be affected by previous surgical scar tissue in the interlaminar approach^17^; and (iii) for elderly patients with disc herniation combined with intervertebral foraminal stenosis, the intervertebral foraminal can be simultaneously expanded and formed by PETD.

In conclusion, we believe that both PEID and PETD are safe, effective, and minimally invasive surgical methods for the treatment of L_5_/S_1_ disc herniation under the conditions that the surgical techniques required have been mastered and the indications for surgery are observed.
